# Development of supraspinatus imaging guidance for primary care physicians with a focus on patient selection

**DOI:** 10.1186/s13089-020-00187-2

**Published:** 2020-09-03

**Authors:** Anurag Dalai, Leanne Langford, Cole Beavis, Haron Obaid

**Affiliations:** 1 Department of Radiology, College of Medicine, University of Saskatchewan, 103 Hospital Drive, Saskatoon, SK S7N 0W8 Canada; 2 Department of Orthopedic Surgery, College of Medicine, University of Saskatchewan, Saskatoon, Canada

**Keywords:** Rotator cuff tendinopathy, Supraspinatus tendinopathy, MRI, Ultrasound, Musculoskeletal

## Abstract

**Background:**

Primary care physicians frequently encounter patients with supraspinatus pathology and face a difficult task of managing this subset of patients using limited imaging resources. The purpose of this study was to develop a guidance that could help primary care physicians choose appropriate imaging tests judiciously for patients with suspected supraspinatus pathology.

**Methods:**

The imaging reports of one hundred patients who underwent ultrasound and MRI for suspected supraspinatus tendinopathy were retrospectively assessed. The supraspinatus tendon was recorded as intact, partial tear (articular or bursal), or full-thickness tear (focal or complete width). The agreement between imaging modalities was then evaluated using factors such as pathology type and age.

**Results:**

There was agreement between modalities in 48/100 patients (Kappa statistic = 0.30). The consistency varied with type of pathology: intact tendons by ultrasound had 55.8% agreement with MRI, partial sided bursal tears 50%, partial sided articular tears 25%, and full-thickness focal tears 33.3%. Full-thickness complete-width tears had a much better agreement with MRI at 90.9%. Age was also significant, with increased disagreement between ultrasound and MRI in patients over 50 years old.

**Conclusions:**

Our data showed that ultrasound findings correlated well with MRI in patients under 50 years of age and also in patients with full-thickness supraspinatus tears. We recommend that primary care physicians may consider using ultrasound as the initial test in younger patients and in patients with suspected full supraspinatus tears, based on clinical exam, with MRI as an option for further evaluation to quantify supraspinatus muscle atrophy. These patient selection recommendations will help promote mindful utilization of scarce resources.

## Background

Shoulder pain is commonly reported in primary care and affects people of all ages [1]. Although the rotator cuff comprises four distinct muscles, tendinopathy in this region most commonly involves the supraspinatus tendon [1]. Proper diagnosis of supraspinatus tendinopathy subsequently allows timely and accurate management. The purpose of this study was to ascertain patient features that will help primary care physicians choose imaging investigations judiciously when considering supraspinatus tendinopathy by evaluating ultrasound and MRI finding agreement.

Primary care physicians rely on history and a complete physical exam to guide further investigation and management. A meta-analysis and systematic review support the incorporation of a thorough history and each of the physical exam components to increase positive predictive value [2]. However, these predictive values can be limited with studies showing varying sensitivity, specificity and positive predictive values regarding individual history and physical exam components [2]. Therefore, clinicians should have a low threshold for diagnostic imaging such as ultrasound and MRI [2]. There is currently no widely agreed upon patient metrics which allow for accurate and timely supraspinatus tendinopathy diagnosis and efficient use of resources [2]. One study showed that a higher age and positive Neer test were important predictors of rotator cuff tear, although the sample size was small [3]. Another study showed certain aspects of the physical exam and history in combination led to the accurate detection of a rotator cuff tear, although only X-ray and ultrasound were used as the imaging modalities [4].

As supraspinatus pathology is extremely common, imaging studies are frequently ordered, causing significant utilization of the healthcare system and its resources. Magnetic resonance imaging (MRI) and ultrasound are both used in the diagnosis of supraspinatus tendinopathy. Previous studies comparing MRI with ultrasound in their diagnostic performance of supraspinatus tears have yielded variable results. A 2013 Cochrane review assessing diagnostic tests in patients with suspected rotator cuff tears in addition to the current American College of Radiology (ACR) appropriateness criteria for suspected rotator cuff tear recommend either ultrasound or MRI as the imaging of choice [5, 6]. Other studies recommend using MRI, with ultrasound as a reasonable initial investigation [7, 8].

Ultrasound studies are generally considered as preliminary tests with MRI follow-up suggested if the initial ultrasound is equivocal or if surgical intervention is being considered [9]. Ultrasound is a very appealing first-line imaging modality for evaluation of tendon pathology as it is widely available, non-invasive, inexpensive, dynamic and has no radiation risk [10, 11]. Recognizing these benefits, it would be helpful for clinicians to have more evidence to choose ultrasound as the initial imaging modality using patient features.

## Methods

Approval for this study was obtained from the research ethics board of our university, and operational approval was granted from our local health region. The study was then carried out in accordance with the approved protocol. The requirement for patient consent was waived during ethical review. This study focused on evaluating diagnostic agreement between ultrasound and MRI for potential supraspinatus tendinopathy and inferring patient characteristics that may aid clinicians in choosing one modality over another. We were unable to compare the accuracy of either ultrasound or MRI studies to operative reports as not all types of supraspinatus tendinopathies are managed surgically.

A retrospective study of 100 patients age 25 and over with suspected rotator cuff pathology who had an MRI of the shoulders in the Department of Radiology at the University of Saskatchewan was performed. The inclusion criteria were any suspected rotator cuff pathology with an ultrasound performed within 3 months of the MRI. The exclusion criteria included a failed exam, previous rotator cuff surgery or the absence of an ultrasound within 3 months of the MRI. One author, a radiology resident, reviewed the ultrasound and MRI reports and categorized the results based on the indicated findings. These reports were reviewed in a retrospective, sequential manner, until a convenience sample of 100 subjects was reached. Images were taken during the period of June 18, 2015 to March 15, 2016. All ultrasound studies were performed by experienced community sonographers and reported by board certified radiologists. These reports were found on the Picture Archiving Communication System (PACS) or through local radiology clinic databases. Findings of each modality focused on the supraspinatus tendon. Results were classified as: intact, partial tear articular, partial tear bursal, full-thickness tear focal or full-thickness tear complete width.

Statistical analyses provided descriptive statistics (means with standard deviations [SD] and frequencies with proportions), both overall and within groups that, respectively, did and did not have agreement between ultrasound and MRI diagnoses. As a goal of the study was to evaluate ultrasound’s ability to predict the MRI result, ultrasound findings and MRI findings were initially cross-classified and the proportions of ultrasounds with an agreeing MRI result were determined, both overall and within each of the five ultrasound diagnostic categories. Kappa statistic was calculated to quantify overall agreement, and Fisher’s exact test was used to determine the presence of differences in agreeing proportions between ultrasound categories. Pairwise comparisons with Bonferroni adjusted *p*-values were subsequently undertaken to identify differing categories. Categories with few cases were combined for additional analysis were clinically and statistically similar, although individual patients were still only deemed to have agreement if the specific ultrasound and MRI diagnoses matched. Comparing characteristics between subjects that did and did not have agreement, age in continuous format was evaluated using the *t*-test; categorical variables were assessed using Chi-square test or Fisher’s exact test. Characteristics were further assessed both individually and combined in respective unadjusted and multivariable logistic regression models to evaluate their ability to predict agreement. Only statistically significant terms (*p* < 0.05) were retained in the final multivariable model. SAS software, version 9.4 (SAS Institute Inc. Cary, NC, USA) was utilized for analysis.

## Results

The ages of the 100 patients included in the study ranged from 25 to 65 years, with an average age of 49.2 years (SD = 10.2 years). The male-to-female ratio was 68 to 32. The MRI results were distributed as 19 full-thickness tears, complete width; 11 full-thickness tears, focal; 13 partial tears, bursal side; 22 partial tears, articular side; and 35 no tear.

There was agreement in the findings between ultrasound and MRI in only 48 out of 100 patients (95% confidence interval 38%, 58%), with a kappa statistic of 0.30. Fisher’s exact testing detected differences in the consistency of agreement between ultrasound and MRI specifically based on the type of pathology identified on ultrasound (*p* = 0.002). Among the 43 patients with the most common ultrasound result, an intact supraspinatus tendon, 24 had the same finding on MRI (24/43 = 55%). Agreement was least consistent for ultrasound-identified partial tears on the articular aspect (7/25 = 25%). In contrast to complete-width, full-thickness tears were nearly all congruent with MRI (10/11 = 90.9%). Agreement proportions for full thickness but focal tears and bursal sided, partial tears were more intermediate and are available in Table 1. Pairwise comparison showed significant differences in agreement when ultrasound diagnosis was a focal, full-thickness tear or an articular sided, partial tear compared to complete width, full-thickness tears (*p* = 0.046 and *p* = 0.002, respectively).

**Table 1:** Distribution of ultrasound results by corresponding MRI findings

Corresponding MRI results	Ultrasound result, *n* (%)					Total
	Intact	Partial tear, articular side	Partial tear, bursal side	Full-thickness tear, focal	Full-thickness tear, complete	
	*n* = 43	*n* = 28	*n* = 6	*n* = 12	*n* = 11	*n* = 100
Intact	*24* (*55.8*)	7 (25.0)	2 (33.3)	2 (16.7)	0 (0.0)	35 (35.0)
Partial tear, articular side	12 (27.9)	*7* (*25.0*)	0 (0.0)	2 (16.7)	1 (9.1)	22 (22.0)
Partial tear, bursal side	4 (9.3)	5 (17.9)	*3* (*50.0*)	1 (8.3)	0 (0.0)	13 (13.0)
Full-thickness tear, focal	2 (4.7)	5 (17.9)	0 (0.0)	*4* (*33.3*)	0 (0.0)	11 (11.0)
Full-thickness tear, complete	1 (2.3)	4 (14.3)	1 (16.7)	3 (25.0)	*10* (*90.9*)	19 (19.0)

Italicized values highlight agreement

Given that data on bursal sided, partial tears and focal, full-thickness tears was relatively sparse and did not convincingly suggest different proportions of agreement between them (Table 1), these patients were further combined with those who experienced articular sided, partial tears to create a larger category of incomplete tears. An ultrasound diagnosis of incomplete tear was again less likely to agree with MRI when compared to complete full-thickness tears (30.4% versus 90.9%, *p* = 0.0008) and also when compared to those viewed as intact on ultrasound (30.4% versus 55.8%, *p* = 0.047).

On univariate assessment (Table 2), agreement of MRI findings with observed ultrasound findings did not significantly differ by sex, although age at or exceeding 50 years and involvement of the right shoulder were initially found to be associated with a greater frequency of disagreement (*p*-value = 0.07 and 0.04, respectively). However, it was also noted that left-sided studies in the sample had ultrasound diagnoses that, as suggested in Table 1, may tend towards better agreement with MRI (i.e. more intact diagnoses and bursal sided, partial tear diagnoses). The right side, in contrast, had more ultrasound articular sided, partial tear diagnoses, the ultrasound diagnosis with the least MRI agreement. Once differences in the type of pathology were controlled for in the multiple regression model, this association became non-significant; laterality was deleted from the final model.

**Table 2:** Comparison of subjects with and without agreement between imaging modalities

	Ultrasound agreement with MRI findings		*p*-value
	Yes, *n* = 48	No, *n* = 52	
Age, mean (SD)	47.9 (10.7)	50.4 (9.6)	0.21
Age, *n* (%)			
Less than 50 years	29 (60.4)	22 (42.3)	0.07
50 years or older	19 (39.6)	30 (57.7)	
Sex			
Male	34 (70.8)	34 (65.4)	0.56
Female	14 (29.2)	18 (34.6)	
Laterality			
Right	20 (42.6)	33 (63.5)	0.04
Left	27 (57.4)	19 (36.5)	
Ultrasound finding			
Intact	24 (50.0)	19 (36.5)	0.0006
Incomplete tear^a^	14 (29.2)	32 (61.5)	
Full-thickness tear, complete width	10 (20.8)	1 (1.9)	

*MRI* magnetic resonance imaging, *SD* standard deviation

^a^Partial thickness tear or full-thickness tear, but focal

**Fig. 1:**
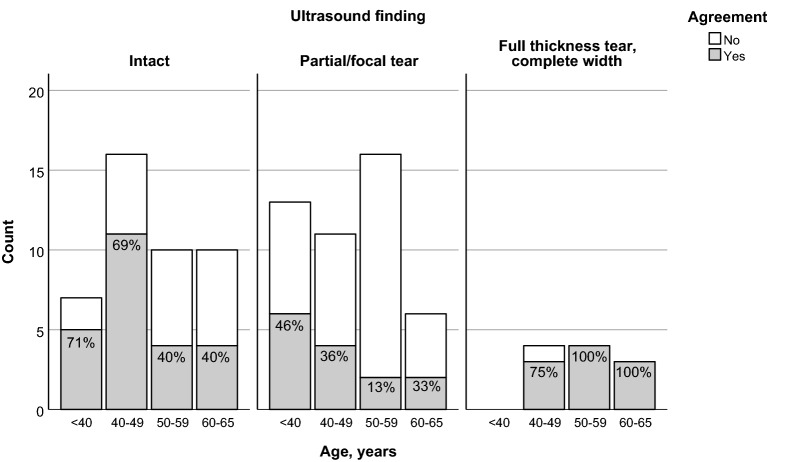
Proportion of patients with agreement by age, stratified by type of ultrasound finding

Notably, a statistically significant difference in diagnostic agreement based on age remained in the adjusted model (Table 3). In older patients, the ultrasound result was less likely to correlate with the MRI study. On average, those 50 years of age and older were 64% (95% CI 12%, 85%) less likely to have their ultrasound result agree with their MRI result compared to younger patients, even after adjustment for type of ultrasound-determined pathology. Agreement as a function of patient age, stratified by type of ultrasound finding, is presented in Fig. 1. Similarly, type of ultrasound finding suggested varying degrees of agreement with MRI depending on the diagnosis, even after adjustment for differences in age. An ultrasound assessment showing an incomplete tear (either partial thickness or complete thickness but focal) was on average 70% (95% CI 26%, 88%) less likely to have the specific corresponding diagnosis on MRI compared to a tendon deemed intact on ultrasound. In contrast, ultrasound-detected full-thickness tears were considerably more likely to have a congruent MRI result (OR − 10.2, 95% CI 1.16, 89.7).

**Table 3:** Simple and multiple logistic regression models predicting ultrasound and MRI agreement

Predictors, simple regression	OR	95% CI	*p*-value
Age, years	0.975	0.937, 1.014	0.21
Age (reference: < 50 years)	0.48	0.22, 1.07	0.07
Sex (reference: female)	1.29	0.55, 3.0	0.56
Laterality (reference: right)	2.3	1.05, 5.3	0.04
Ultrasound finding (reference: intact)			
Incomplete tear^a^	0.35	0.15, 0.83	0.02
Full thickness	7.9	0.93, 67.4	0.06
Predictors, multiple regression			
Age (reference: < 50 years)	0.36	0.15, 0.88	0.03
Ultrasound finding (reference: intact)			
Incomplete tear^a^	0.30	0.12, 0.74	0.009
Full thickness	10.2	1.16, 89.7	0.04

*MRI* magnetic resonance imaging, *OR* odds ratio, *CI* confidence interval

^a^Partial thickness tear or full-thickness tear, but focal

## Discussion

Imaging plays a vital role in diagnosing musculoskeletal disease or injury. Currently there are various modalities that can help a clinician such as ultrasound, MRI, plain film, fluoroscopy, and CT. With regard to rotator cuff injuries, ultrasound and MRI imaging are the two most commonly used modalities. When rotator cuff pathology is suspected an ultrasound is most often the first choice whether in the community or in the hospital depending on the acuity [9]. A systematic review by Liang et al. concluded that ultrasound is highly efficient in assessing supraspinatus tears due to its high sensitivity, specificity and accuracy [12]. However, a network meta-analysis by Liu compared the diagnostic values of MRA, MRI and ultrasound which showed that high-field MRA had the highest diagnostic value [13]. The decision to proceed to MR imaging usually depends on whether the ultrasound result is equivocal or if the clinical history and exam suggest further investigation or if surgery is being considered [9].

The results of this study showed that the agreement between ultrasound and MRI regarding supraspinatus tendinopathy evaluation varied. Despite the general variability in modality correlation, ultrasound diagnosis with full-thickness complete-width tears had the best agreement at 90.9% with MRI. The next best agreement, an ultrasound diagnosis of intact tendon, shared only 55.8% agreement with MRI. This is in keeping with the literature which showed that both ultrasound and MRI have excellent diagnostic accuracies regarding full-thickness tears (sensitivity of 92% and 94%, respectively, and specificity of 93%) [14]. The evaluative agreement also appeared less consistent with increasing age, at least among ultrasound findings of intact tendons and partial tears. This may, in part, be related to older patients’ inability to cooperate with ultrasound scan techniques due to restricted range of shoulder motion in this patient population.

Based on our data, there was only fair agreement between the two modalities. Our results showed that ultrasound findings generally had poor correlation with MRI findings across most types of supraspinatus tendinopathy which contradicts many studies including a systematic review that stated ultrasound correlates with MRI 95% of the time for rotator cuff tendinopathy [15]. Our study’s sample size may be in part a reason for this. An example of this discrepancy from our study was in one patient where a supraspinatus tendon appears to be intact on an ultrasound study, but further investigation with MRI showed a high-grade partial thickness tear. If a complete tear is suspected, ultrasound may be a good first-line imaging modality as the findings correlated well with MRI imaging. However, if the ultrasound is negative and there is ongoing clinical concern, MRI should be considered which is keeping in line with current algorithms used for rotator cuff imaging [8]. Although ultrasound has similar sensitivity and specificity compared to MRI when diagnosing some types of supraspinatus tendinopathy, MRI is shown to be better at accurately depicting the lesion size, medial retraction and degree of muscle fatty infiltration [16]. Partial thickness tears were where most of the discrepancy between ultrasound and MRI findings were in our study. Pertaining to partial thickness tears, one study showed that MRI is not completely reliable [2]. Ultrasound and MRI both have poor sensitivity (52% and 74%, respectively) in diagnosing partial thickness tears, but otherwise have no statistically significant difference in diagnosing total ruptures [17]. These findings are similar in re-current tears [18]. In order to improve the diagnosis of partial thickness tears, dynamic ultrasound in combination with MRI was suggested [2].

When it comes to patient and clinical factors, there is currently little in the literature discussing the correlation of rotator cuff physical exam findings with findings on various imaging modalities or arthroscopy and eventual diagnosis. Therefore, it can be difficult for a clinician to choose between ultrasound and MRI. One study with a small sample size showed that clinical patient shoulder examinations had moderate diagnostic value for diagnosing rotator cuff tendinopathy when compared to the patient’s respective magnetic resonance arthrogram (MRA) studies [2]. Higher age and a positive Neer test were found to be the most important predictors of rotator cuff tears [2]. Another study found combinations of certain aspects of the history and physical exam led to a greater diagnostic accuracy of rotator cuff tears [3]. Neither of these studies, however, used both ultrasound and MRI nor were components of the physical exam or history found to help guide clinicians in choosing one imaging modality over another.

There were a few limitations to this study. Firstly, the study is retrospective in nature, and included only those individuals who underwent both forms of imaging. However, our convenience sample was sequential and included all patients who met our inclusion and exclusion criteria during a relatively long period of observation; thus, we believe our findings to be adequately representative of this specific group. The noted associations require further confirmation via prospective work. Secondly, the types of ultrasound machines and the settings that sonographers used to conduct the scans were not analysed as ultrasound is widely known to be an operator-dependent imaging test and settings may vary between different operators. Thirdly, interobserver differences that could exist between radiologists who interpreted the ultrasound and MRI could not be evaluated due to the retrospective nature of the study. Fourthly, radiologists who interpreted the MRI scans were not blinded to the ultrasound reports. Fifthly, our findings arise from a single location, which potentially limits generalizability. Sixth, although we anticipate that the appropriate use of ultrasound over MRI would result in immediate cost-saving, a full cost–benefit analysis is beyond the scope of this paper. Furthermore, we focused on the supraspinatus tendon as it is the most commonly affected which usually requires surgical repair. These results cannot be extrapolated when considering the diagnosis of other rotator cuff tendon injuries.

## Conclusion

There was considerable variability between ultrasound and MRI in the assessment of supraspinatus tears. Our data showed that ultrasound studies among younger patients correlate better with MRI than patients over 50 years of age where disagreement increases. Primary care physicians can confidently consider using ultrasound as the initial test in younger patients and in patients with suspected full supraspinatus tears or intact tendons based on clinical exam with MRI as an option for further evaluation if necessary. In the case of older patients or in patients with physical exam findings that are equivocal that may not suggest a complete tear nor intact tendon, ultrasound or MRI may be preferred. However, with studies suggesting that neither ultrasound and MRI are particularly reliable nor sensitive for partial tears, ultrasound may serve as a reasonable first step for economic and patient accessibility reasons. These patient selection recommendations would help promote mindful resource utilization, keeping in mind the economic benefits of ultrasound over MRI.

## Data Availability

Data can be made available upon request but will be stripped of all personal identifiers to comply with ethical conduct of human experimentation.
